# Perspective and Therapeutic Potential of the Noncoding RNA–Connexin Axis

**DOI:** 10.3390/ijms25116146

**Published:** 2024-06-02

**Authors:** Xinmu Li, Zhenzhen Wang, Naihong Chen

**Affiliations:** State Key Laboratory of Bioactive Substances and Functions of Natural Medicines, Institute of Materia Medica & Neuroscience Center, Chinese Academy of Medical Sciences and Peking Union Medical College, Beijing 100050, China; lixinmu@imm.ac.cn

**Keywords:** noncoding RNA, microRNA, lncRNA, connexin, disease

## Abstract

Noncoding RNAs (ncRNAs) are a class of nucleotide sequences that cannot be translated into peptides. ncRNAs can function post-transcriptionally by splicing complementary sequences of mRNAs or other ncRNAs or by directly engaging in protein interactions. Over the past few decades, the pervasiveness of ncRNAs in cell physiology and their pivotal roles in various diseases have been identified. One target regulated by ncRNAs is connexin (Cx), a protein that forms gap junctions and hemichannels and facilitates intercellular molecule exchange. The aberrant expression and misdistribution of connexins have been implicated in central nervous system diseases, cardiovascular diseases, bone diseases, and cancer. Current databases and technologies have enabled researchers to identify the direct or indirect relationships between ncRNAs and connexins, thereby elucidating their correlation with diseases. In this review, we selected the literature published in the past five years concerning disorders regulated by ncRNAs via corresponding connexins. Among it, microRNAs that regulate the expression of Cx43 play a crucial role in disease development and are predominantly reviewed. The distinctive perspective of the ncRNA–Cx axis interprets pathology in an epigenetic manner and is expected to motivate research for the development of biomarkers and therapeutics.

## 1. Introduction

Noncoding RNAs (ncRNAs) consist of microRNAs (miRNAs), transfer RNA (tRNA)-derived small RNAs (tsRNAs), PIWI-interacting RNAs (piRNAs), and long noncoding RNAs (lncRNAs); among lncRNAs, distinct subtypes have been identified, such as pseudogene-derived lncRNAs and circular RNAs (circRNAs) [1]. Although ncRNAs do not encode proteins, they are involved in essential biological processes, and their dysregulation contributes to various diseases. The overexpression of miRNAs in cancers can function as oncogenes and promote cancer development by negatively regulating tumor suppressor genes and/or genes that control cell differentiation or apoptosis, whereas the underexpression of miRNAs in cancers can function as tumor suppressor genes that can inhibit cancers by regulating oncogenes and/or genes that control cell differentiation or apoptosis [1]. Several ncRNAs have been identified to participate in atherogenesis during the development of atherosclerosis [2]. Through epigenetic mechanisms, ncRNAs affect neurogenesis, synaptic plasticity, and neurotransmitter systems to regulate depression [3,4]. Owing to the close relationship between ncRNAs and diseases, therapeutic approaches targeting ncRNAs have received growing interest and are considered promising [5,6,7].

Human genes encode 21 kinds of connexin (Cx) proteins comprising four transmembrane domains, two extracellular loops, and one cytoplasmic loop [8]. Six connexins form a hemichannel termed a connexon [8]. Connexons that dock between the membranes of two adjacent cells form intercellular channels termed gap junctions, which aggregate into plaques [8]. Gap junctions allow the transfer of ions and small molecules across cell membranes and regulate intercellular communication, cell differentiation, and cell proliferation [9]. Hemichannels mediate the transfer of small molecules between the cytoplasm and extracellular environment and primarily function under pathological conditions [10]. Connexin gene mutations and connexin dysregulation are implicated in central nervous system (CNS) disorders and cardiovascular diseases [11,12,13]. Among all connexins, connexin 43 (Cx43) is the most studied, and potential therapeutic approaches based on Cx43 have been widely implicated [14,15,16]. (See Table S1 for a summary of connexin genes and corresponding proteins.)

**Figure 1:**
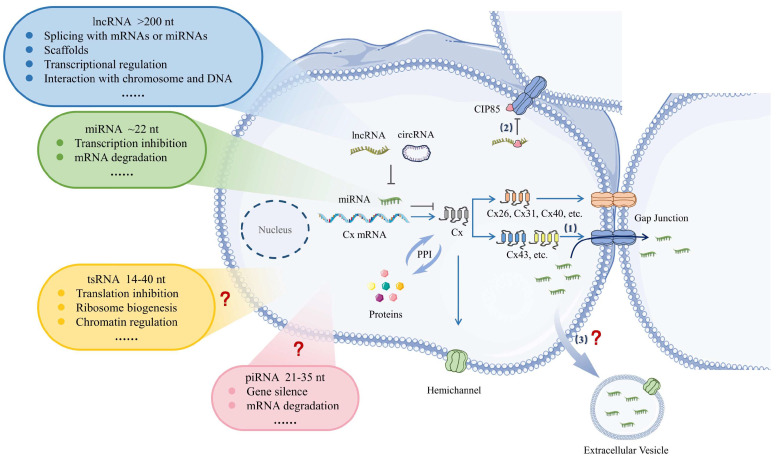
Diagram of the ncRNA–Cx axis. miRNAs act as translation suppressors by inhibiting transcription or inducing mRNA degradation. miRNAs bind to the 3′ untranslated regions of connexin mRNAs, preventing the Cx mRNAs from being translated, thereby contributing to disease development. lncRNAs, including circRNAs, act as sponges to abolish miRNA function, abrogating the miRNA-induced inhibition of Cx mRNAs. Moreover, miRNAs and lncRNAs interact with various proteins or serve as scaffolds to influence Cx function and distribution. tsRNAs and piRNAs regulate gene expression; however, how they regulate Cx remains unclear. Notably, (1) gap junctions particularly assembled from Cx43 provide pathways for the intercellular transport of miRNAs between cancer cells; (2) lncRNA CCRR’s reaction with CIP85 interrupts the binding of CIP85 and Cx43, thereby preventing the removal of Cx43 from the myocardial cell membrane; and (3) extracellular vesicles equipped with hemichannels have been implicated in the delivery of miRNAs to distant cells. However, supporting evidence for this mechanism is currently limited. The interaction between tsRNAs, piRNAs, and connexins needs to be further investigated. Cx, connexin; lncRNA, long noncoding RNA; circRNA, circular RNA; miRNA, microRNA; tsRNAs, transfer RNA (tRNA)-derived small RNAs; piRNAs, PIWI-interacting RNAs; nt, nucleotide; PPI, protein–protein interaction.

In this review, we focus on ncRNA–Cx axis dysregulation in diseases and pathological processes, as well as ncRNA delivery through gap junctions. Diseases are stratified according to organ systems, and the ncRNA–Cx axis summarized in this review has been documented in scientific publications over the past five years. miRNAs directly bind to the mRNAs of connexins, inhibiting mRNA translation. In addition, lncRNAs or circRNAs can be spliced with miRNAs to interrupt the effects of miRNAs. Moreover, ncRNAs interact with proteins to regulate Cx function. The mechanisms involved in the ncRNA–Cx axis are shown in Figure 1.

## 2. Disorders Modulated by Noncoding RNAs through Relevant Connexins

### 2.1. Disorders in the CNS

The ncRNAs presented herein may regulate connexin expression or participate in the modification of connexins. Recent studies have implicated the expression and modification of connexins in depression, brain injury, neurodegenerative diseases, and numerous pathophysiological mechanisms, including apoptosis, neuroinflammation, and cellular proliferation and migration.

Gap junctions assembled from Cx43 play vital roles in maintaining astrocyte networks and consequently coordinate neuronal activity and synaptic transmission [17,18]. The downregulation of Cx43 reduces gap junction coupling, which alters the transmission of monoamines, glutamate, calcium, ATP, and cAMP and affects neuronal activity and synaptic function [19]. Decreased Cx43 expression and gap junction dysfunction in an animal model of depression were reversed by antidepressant treatment [20]. Moreover, astrocytic Cx43 is widely involved in CNS regulation, contributing to gap junction dysfunction and neuroinflammation in depression [16]. In a study using an unpredictable chronic mild stress (UCMS)-induced mouse model of depression, miR-221/222 directly bound to the 3′ untranslated regions (3′ UTR) of Cx43 mRNA and downregulated Cx43 protein levels; this could be reversed by genistein treatment, subsequently ameliorating depression-like behaviors in the mice [21]. miR-205 also targeted the *Gja1* gene, which encodes the Cx43 protein [22]. The administration of Mahonia alkaloids reduced miR-205 content and consequently improved Cx43 levels, alleviating depression in reserpine-treated rats [22]. miR-106a was confirmed to bind to the 3′ UTR of Cx43 mRNA, and an miR-106a antagonist could repress apoptosis, alleviate oxidative stress, and enhance Cx43 protein content in glucose oxidase-treated rat cochlear marginal cells [23]. Furthermore, in gentamicin-induced rat models, the miR-106a antagonist exerted therapeutic effects on the pathological state of sensorineural hearing loss by regulating Cx43 [23].

Although not an immediate binding target, miR-182 inhibition tends to reverse Cx43 degradation in injured astrocyte cultures, indicating that miR-182 may decrease Cx43 expression [24]. Cx43 has been phosphorylated by extracellular-signal-regulated kinase (ERK)1/2 in rats subjected to cerebral ischemia [25]. In vitro models have shown that the overexpression of the miR-302/367 cluster has suppressed the phosphorylation of both ERK1/2 and Cx43, and in vivo studies revealed that elevated expression of the miR-302/367 cluster improved cognitive function and neuronal damage in rats after traumatic brain injury [26]. These findings suggest that the miR-302/367 cluster regulates ERK signaling to inhibit Cx43 phosphorylation and mitigates brain damage [26]. Jullienne et al. found that aquaporin 4 (AQP4) and Cx43 were significantly decreased after intracortical injection of siAQP4, and miR-224 and miR-19a were significantly increased [27]. They reported that this may be responsible for losses in astrocyte connectivity and tissue water mobility [27]. These off-target effects of siAQP4 suggest that miR-224 and miR-19a may be involved in amyotrophic lateral sclerosis, which shows concomitant and aberrant overexpression of AQP4 and Cx43 [27,28].

lncRNAs also function as the regulators of several CNS diseases [29,30]. lncRNA Pnky has a modulatory effect on Cx43 during neural stem cell migration [31]. lncRNA Pnky overexpression has enhanced the protein levels of some critical regulators of neural stem cell migration, including Cx43, whereas lncRNA Pnky depletion has decreased them in vitro. Consistently, cells overexpressing lncRNA Pnky transplanted into mice exhibited greater migration distances in the spinal canal than those in which lncRNA Pnky was depleted [31]. Knockdown of lncRNA NEAT1 downregulated Cx32 expression via the promotion of miR-1301-3p, a suppressor of *Gjb1* (Cx32); suppressed the α-synuclein-induced inflammatory response; and reduced apoptosis in a cellular Parkinson’s disease (PD) model [32]. Abnormal α-synuclein aggregation contributes to neuroinflammation and is a key player in the pathology of PD [33]. This miR-1301-3p/*Gjb1* signaling pathway, which is regulated by lncRNA NEAT1, offers potential therapeutic targets for PD treatment.

However, Cx43 enrichment does not always provide benefits. In cultured γ-radiation-induced astrocytes, miR-206 significantly lowered Cx43 expression and inflammatory cytokine release. In contrast, the Cx43-plasmid had the opposite effect, revealing that miR-206 relieves neuroinflammation by downregulating Cx43 [34]. Astrocytes transfected with miR-301a-3p, which can bind to Cx43 and downregulate its expression, have exhibited increased proliferative potential and decreased apoptosis; however, astrocytes overexpressing the Cx43 protein have exhibited high cell death rates [35].

Cx43 overexpression is widely observed in the inflammation, apoptosis, and proliferation associated with cancer cells. The detrimental role of Cx43 may originate from its channel-dependent and channel-independent functions. Cell death could be caused by gap junction-mediated bystander killing [36]. In addition, although hemichannel-mediated diseases have not yet been proven, Caruso et al. reviewed the molecular mechanisms of Cx43 hemichannel opening and their contribution to neuroinflammation and oxidative stress [37]. Activation of the inflammasome complex, NF-κB signaling pathways, and alterations in intracellular calcium levels can induce the opening of Cx43 hemichannels, thereby facilitating the release of ATP [37]. Dysregulated secretion of ATP and other gliotransmitters through Cx43 hemichannels disrupts CNS homeostasis [37]. The non-canonical functions of Cx43 include participation in gene transcription, development, and scaffolding complexes, as well as facilitating glioma invasion via a channel-independent pathway [38,39]. The conundrum of connexins or gap junctions serving as protectors or risk factors is determined by intercellular signaling [40].

Connexins have long been considered drug targets for neurological and neuropsychiatric disorders [17]. As shown in Table 1, recently established interactions between ncRNAs and connexins may augment the array of therapeutic strategies available for managing CNS diseases.

### 2.2. Disorders in the Cardiovascular System

Cx43 is the most abundant and extensively studied connexin in the cardiovascular system. Downregulated and altered distribution of Cx43 levels have been observed in the pathologies of several heart diseases [13]. Studies involving antisense peptides that knock down Cx43 expression or block Cx43 channels to alleviate ischemia/reperfusion injury have demonstrated the adverse effects of Cx43 [16,41]. Recently, ncRNAs were implicated in the regulation of connexins in the abovementioned phenomena. miR-1, miR-206, and lncRNAs are highly correlated with the regulation of Cx43, thereby participating in cardiovascular disorders [42,43,44].

For example, Cx43 may be a key target of miR-1 in the regulation of cardiovascular diseases. A recent study has identified the potential binding sites of miR-1 in the 3′ UTR of Cx43 [42]. The overexpression of miR-1 in mouse cardiomyocytes induced atrioventricular blocks accompanied by reduced Cx43 protein expression [45]. Under oxidative stress, miR-1 levels increase and Cx43 expression decreases [46,47]. The downregulation of Cx43 was also correlated with increased miR-1 levels in in vitro models of ischemia/hypoxia, and treatment with miR-1 inhibitors elevated Cx43 expression [48,49]. Furthermore, an endothelial dysfunction model induced by the transfection of miR-1 exhibited reduced Cx43 protein levels [50].

miR-206 is associated with arrhythmia and thrombosis by directly binding to Cx43 mRNAs, resulting in decreased Cx43 protein levels [43]. Conversely, inhibiting miR-206 has upregulated Cx43 levels and alleviated ischemia–reperfusion arrhythmia [43]. Consistent with these findings, miR-206 overexpression caused cardiac arrhythmia in mice by downregulating Cx43 [51]. miR-206 modulates cellular autophagy by targeting Cx43 mRNA [52]. The upregulation of miR-206 reduces Cx43 expression and increases autophagy-related proteins; *Gja1* silencing exerts the same effects [52].

The involvement of other miRNAs and lncRNAs in cardiovascular disease is summarized in Table 2. Not only a direct target, Cx43 is a downstream factor modulated by the ncRNA–protein/miRNA axis. Signaling factors, including the platelet-derived growth factor [53] and Wnt3a [54], regulate Cx43 via miRNAs. Notably, ncRNA/protein complexes also have extensive functions. lncRNA CCRR binds to the Cx43-interacting protein CIP85 and prevents the endocytic trafficking of Cx43, thereby controlling the membrane distribution of Cx43 and gap junction functioning [44]. Similarly, lncRNA Gm2694 interacts with the chaperone protein GRP78 to trigger an unfolded protein response, which is associated with endoplasmic reticulum disruption [55]. miRNAs that interact with proteins are involved in the crucial mechanism of RNA splicing [56].

### 2.3. Disorders in Other Systems

Cx43 upregulation is associated with the inflammatory response and apoptosis observed during the development of osteoarthritis (OA), intervertebral disc degeneration, and ossification [64,65,66,67]. In vitro, chondrocytes induced by interleukin-1β have exhibited Cx43 overexpression, which has facilitated the upregulation of Toll-like receptor 4 (TLR4), myeloid differentiation primary response 88 (MyD88), and nuclear factor κB (NF-κB) [65]. Simultaneously, miR-382-3p could suppress Cx43 and inhibit this TLR4/MyD88/NF-κB signaling pathway [65].

Ferroptosis is involved in the pathology of OA and differs from apoptosis and autophagy [68]. Cx43 overexpression aggravates ferroptosis in chondrocytes, a process rescued by miR-1 [69]. Excess Cx43 triggered by a knockdown of miR-1 could also form hemichannels that can promote the extracellular release of ATP [70]. Osteogenesis requires Cx43 expression, and upstream ncRNAs that regulate Cx43 have been identified [67,71].

Connexin26 (Cx26) and connexin30 (Cx30) form heteromeric and heterotypic gap junctions in the cochlea, and the mutations of Cx26 are responsible for congenital hearing loss [72,73]. miR-34c-5p directly binds to Cx26 mRNAs and downregulates their expression [74]. Instead of inhibiting upstream ncRNAs first, Gentile et al. knocked out Cx30 in a mouse model and investigated the miRNAs whose expressions were altered [74]. They identified the upregulated miRNAs and downstream targets linked to hearing loss [74]. This method shows that the dysregulation of connexins can cause abnormal ncRNA expression, leading to pathological processes. The ncRNAs and related connexins associated with pathological processes are summarized in Table 3.

### 2.4. Several Kinds of Cancer

Cell proliferation and migration in lung cancer [76], bladder cancer [77,78], and hypopharyngeal squamous cell carcinoma [79] can be suppressed by the overexpression of certain ncRNAs along with decreased Cx43 levels. This indicates the crucial role of Cx43 in the development of cancer and tumor metastasis [79,80,81]. In contrast, miRNAs are upregulated and connexins are downregulated in ovarian cancer [82], leukemia [83], gastric cancer [84], pancreatic cancer [85,86], and melanoma [87,88], suggesting an inhibitory effect of Cx43 on cancer progression.

In addition to the biochemical perspective of the ncRNA–Cx axis in cancer development, research on tumor drug resistance is worth investigating. Non-small-cell lung cancer (NSCLC) cells are more sensitive to cisplatin due to the overexpression of miR-613, and this enhanced chemosensitivity can be partially reversed by the upregulation of *Gja1* [76]. This decreased drug resistance is partially owing to the lack of Cx43 and few hemichannels that may transport drug molecules out of cells [70]. However, the overexpression of miR-206 and subsequent downregulation of Cx43 enhance cisplatin resistance in ovarian cancer cells [82]. The conflicting reports on the effects of Cx43 on chemoresistance above are partly attributed to the multiple targets of miRNAs in addition to connexins. Furthermore, other molecules are implicated in this phenomenon [89,90], and the roles of connexins are tissue-specific [73]. The ncRNAs and related connexins that were identified during investigations of cancer development are summarized in Table 4.

## 3. The Transfer of Noncoding RNAs through Gap Junctions

In 2011, Lim et al. reported that miR-127, -197, -222, and -223 could be transported via gap junctions from the bone marrow stroma to breast cancer cells to modulate the cell cycle [93]. This phenomenon is considered one of the key discoveries showing that gap junctions directly regulate cancer [40]. Lemcke et al. summarized the co-culture systems that deliver miRNAs through gap junctions (composed of Cx43 or other connexins) in vitro [94]. The co-culture systems encompass mesenchymal stem cells, macrophages, glioma cells, cardiomyocytes, and astrocytes as donor or recipient cells for certain miRNAs [94,95]. Similar heterocellular Cx43-dependent pathways and miRNA transfer were observed in co-cultured melanoma cells and T lymphocytes [96]. Valiunas et al. transfected miR-16 and small interfering RNAs (siRNAs) into stem cells, which were then used to transfer these toxic oligonucleotides into three cancer cell lines via gap junctions to inhibit cell proliferation or induce cell death [97]. These facts have persuaded researchers to study the molecular mechanisms underlying these phenomena, particularly the molecular diameters of oligonucleotides and energy supply. Lemcke et al. proposed possible molecular mechanisms for miRNA shuttling via gap junctions based on experimental and computational data, suggesting that the transfer of miRNAs requires the assistance of the miRNA-binding protein Argonaute (AGO) and non-AGO proteins [98]. However, the present models of gap junction-dependent miRNA transport remain speculative, and novel miRNA-binding proteins must be identified to clarify the underlying mechanisms of gap junction-mediated miRNA transport [98].

In addition to mediating the transmission of miRNAs between adjacent cells, connexins also participate in extracellular vesicle (EV) functions. Cx43 has been detected in the EVs released from multiple human cell lines cultured in vitro [99]. Martins-Marques et al. isolated Cx43-containing EVs from human plasma and proposed that Cx43 participates in miRNA selection, forming functional channels at the EV surface that deliver miRNAs containing stable secondary structural elements to distant cells [100,101]. Similarly, Acuña et al. confirmed that Cx46-containing EVs could be released by breast cancer cells and proposed that Cx46 in the membranes of EVs facilitates the docking and internalization of the receptor cells [102]. Whether the miRNAs contained in EVs can be delivered to remote cells via hemichannel pairing requires further investigation.

## 4. Therapeutic Potential of the ncRNA–Cx Axis

ncRNA biomarkers and RNA interference (RNAi) technology have gained significant traction in clinical research. ncRNA profiles primarily serve as biomarkers to reveal predisposition to disease. During different stages of multiple sclerosis (MS), specifically clinically isolated syndrome (CIS) and relapsing–remitting MS (RRMS), serum EV miRNAs were significantly dysregulated [103]. Notably, CIS-specific serum EV miRNAs were downregulated, whereas RRMS-specific serum EV miRNAs were upregulated [103]. These MS stage-specific EV-derived miRNAs have potential predictive value [103]. Circulating miRNA profiles show features distinguishing transient ischemic attack (TIA) from acute ischemic stroke (AIS) [104]. Eleven miRNAs were differentially expressed in the sera of patients with TIA and AIS, which could help discriminate between these two diseases [104]. Clinical trials of ncRNA therapeutics have exploited RNAi technology to directly suppress target mRNA. Recently, siRNAs olpasiran [105] and lepodisiran [106], targeting lipoprotein synthesis, have emerged as effective treatments for cardiovascular disease. The siRNA agent lumasiran was the first FDA-approved prescription for primary hyperoxaluria type 1 that targets glycolate oxidase [107]. However, compared to the encouraging progress of ncRNAs and siRNAs in clinical practice, the clinical application of connexins is limited. Despite the association between connexin dysregulation and various diseases, no molecules targeting connexins are currently being tested in clinical trials.

To bridge the gap between the essential biological roles of connexins and their limited clinical utility, we suggest that connexins can be regulated by modulating the ncRNAs upstream or downstream of the connexins. Cx43 plays a key role in neuroinflammation [108,109]. Antagomirs to miR-221/222 [21], miR-205 [22], and miR-224 [27] can upregulate Cx43 protein levels in glial cells and have anti-neuroinflammatory potential. Carefully designed siRNAs and antisense oligonucleotides can suppress upstream ncRNAs, such as lncRNA ANRIL [61] to rescue Cx43 or to reduce Cx43 levels by suppressing lncRNA CCRR [44] and lncRNA MALAT1 [62]. More directly, CRISPR-Cas9 technology can be employed to edit ncRNAs, such as lncRNA Pnky, to regulate connexin gene expression. In addition, the use of EVs as drug delivery systems to target the ncRNA–Cx axis is an intriguing strategy. Cx43 can form channels on the surface of EVs and allow drug molecules to pass through [110]. Inspired by this phenomenon, we propose that Cx43 hemichannels can be embedded in artificial EV surfaces to facilitate the delivery of molecules targeting the ncRNA–Cx axis. The abovementioned oligonucleotides, ncRNA-targeted small molecules [111], and adeno-associated virus vectors carrying connexin genes are desirable cargos for these EVs. These EV drug carriers can enhance the uptake efficiency and infiltration of oligonucleotides into the blood–brain barrier, providing a promising approach for treating cancers and CNS diseases [97,112,113].

## 5. Conclusions and Perspectives

In the past few years, researchers have published exceptional reviews on ncRNAs [6,114] and connexins [115,116]; however, summaries on the ncRNA–connexin axis are inadequate. Herein, we have compiled examples published in the past five years that illustrate how connexins are modulated by relevant ncRNAs and how this interaction is associated with the development of many diseases.

Generally, miRNAs bind to the 3′ UTR of the connexin mRNA and interfere with mRNA translation, blocking connexin protein synthesis. This pathological process dysregulates the miRNA levels, leading to a negative correlation with connexin levels. lncRNAs and circRNAs act as sponges that inhibit miRNA functions. Therefore, downstream connexin levels are positively correlated with lncRNAs and circRNAs. Although less common, cases in which the connexin (Cx43) mRNA is located upstream of the miRNAs have been reported [117,118]. Indirectly, ncRNAs regulate or interact with the proteins upstream of connexins, thereby facilitating pathological processes. In particular, ncRNAs can be transported through gap junctions, particularly Cx43, which transfers ncRNAs between neighboring cells in vitro. Cx43 has been proposed to constitute EVs that deliver ncRNAs to remote tissues; however, this mechanism requires further investigation.

However, this study has some shortcomings. Owing to complex protein–protein interactions and tissue specificity, the underlying mechanisms through which connexins and ncRNAs contribute to disease development have not been clearly explained. Notably, developing therapies that regulate the ncRNA–Cx axis is challenging. First, researchers should be aware that, because of limited clinical samples and variations in research methodologies, the selected ncRNA profiles may exhibit limited repeatability, which is directly associated with the selection of ncRNAs to be further studied [119]. Second, in addition to their role as gap junctions, connexins engage in intricate downstream protein–protein interactions [39]. Therefore, the regulation of the ncRNA–Cx axis may potentially result in off-target effects. Third, although many in vitro studies on the intercellular transport of miRNAs through gap junctions have been reported, in vivo research is lacking. Therefore, it is important to acknowledge that utilizing EVs with Cx43 channels for the delivery of oligonucleotides or small molecules is still at a conceptual stage because transferring oligonucleotides through gap junctions requires a substantial energy supply. In the future, researchers should attempt to elucidate the molecular mechanisms before and after the ncRNA–connexin axis to pave the way for efficacious therapies.

## Figures and Tables

**Table 1:** Noncoding RNAs and related connexins involved in CNS diseases.

Animal Tissue/Primary Cells	Cell Line	Noncoding RNAs	Molecular Target	Related Connexin	Disease/Pathology	Ref
Prefrontal cortexes of mice	Human U87-MG astrocytes	miR-221/222 ↑	Cx43 ↓	Cx43 ↓	Depression	[21]
Prefrontal cortexes and hippocampuses of rats	Human glioma cells U251	miR-205 ↑	Cx43 ↓	Cx43 ↓	Depression	[22]
Rat cochlear marginal cells	/	miR-106a ↑	Cx43 ↓	Cx43 ↓	Cell apoptosis	[23]
Cortexes of mouse brains/mouse cortical astrocytes	/	miR-182 ↑	Cortactin ↓	Cx43 ↓	Stroke	[24]
/	Human neuroblastoma SH-SY5Y cells	miR-302/367 ↓	p-ERK1/2 ↑	p-Cx43 ↑	Traumatic brain injury	[26]
Cortexes of rat brains/mouse cortical astrocytes	/	miR-224 ↑	Cx43 ↓	Cx43 ↓	Astrocyte connectivity decrease	[27]
/	HA-1800 normal astrocytes	miR-206 ↓	Cx43 ↑	Cx43 ↑	Neuroinflammation	[34]
Cortex of rat brain/rat cortical astrocytes	/	miR-301a-3p ↓	Cx43 ↑	Cx43 ↑	Cell apoptosis	[35]
Spinal cord of mice	Murine neural stem cells C17.2 and NE4C	lncRNA Pnky ↑	U2AF1	Cx43 ↑	Cell migration	[31]
/	Human neuroblastoma SH-SY5Y cells	lncRNA NEAT1 ↑	miR-1301-3p ↓	Cx32 ↑	Cell apoptosis	[32]

↑: upregulation; ↓: downregulation. U2AF1: U2 small nuclear RNA auxiliary factor 1.

**Table 2:** Noncoding RNAs and related connexins involved in cardiovascular disorders.

Animal Tissue/Primary Cells	Cell Line	Noncoding RNAs	Molecular Target	Related Connexin	Disease/Pathology	Ref
Cardiac tissue of mice	HL-1 cells	miR-1 ↑	Cx43 ↓	Cx43 ↓	Viral myocarditis	[42]
Cardiac tissues of mice/mouse ventricular cardiomyocytes	/	miR-1 ↑	Cx43 ↓	Cx43 ↓	Cardiac conduction delay	[45]
Rat cardiomyocytes	/	miR-1 ↑	Cx43 ↓	Cx43 ↓	Myocardial infarction	[46]
/	iPSC-CMs, HL-1 cells	miR-1 ↑	Cx43 ↓	Cx43 ↓	Arrhythmia	[47]
Cardiac tissue of rats	H9c2	miR-1 ↑	Cx43 ↓	Cx43 ↓	Ischemia/reperfusion injury	[48]
Rat VSMCs	/	miR-1 ↑	Cx43 ↓	Cx43 ↓	Hypoxia	[49]
Rat PASMCs	/	miR-1 ↑	Cx43 ↓	Cx43 ↓	Endothelial dysfunction	[50]
Cardiac tissue of mice	/	miR-206 ↑	Cx43 ↓	Cx43 ↓	Arrhythmia	[43]
Cardiac tissue of mice	/	miR-206 ↑	Cx43 ↓	Cx43 ↓	Arrhythmia	[51]
Vascular tissues of mice	EPCs	miR-206 ↑	Cx43 ↓	Cx43 ↓	Autophagy	[52]
Myocardial tissue of rats/rat myocardial cells	293T cells	miR-23a ↑	Cx43 ↓	Cx43 ↓	Ischemia/reperfusion injury	[57]
Human cardiac fibroblast cells	/	miR-125b-5p ↑	Cx43 ↓	Cx43 ↓	Cardiac fibrosis	[58]
Heart tissue of mice	Rat myocardial fibroblast cells	miR-218-5p ↓	Cx43 ↑	Cx43 ↑	Myocardial fibrosis	[59]
/	HL-1 cells	miR-199a+miR-22 ↑	Cx40 ↓	Cx40 ↓	Atrial fibrillation	[60]
Atrial tissue of rats	/	miR-29b-3p ↓	PDGF-B ↑	Cx43 ↓	Atrial fibrosis	[53]
Atrial tissue of rats	/	miR-27b-3p ↓	Wnt3a ↑	Cx43 ↓	Atrial fibrillation	[54]
Mouse ventricular myocytes	AC16 cells	lncRNA CCRR ↓	CIP85 ↑	Cx43 ↓	Cardiac conduction block	[44]
Human cardiomyocytes	/	lncRNA ANRIL ↑	*CDKN2A* ↓	Cx43 ↓	Cardiac fibrosis	[61]
Rat aortic endothelial cell	293T cells	lncRNA MALAT1 ↑	miR-30c-5p ↓	Cx43 ↑	Atherosclerosis	[62]
Right atrial appendages of humans	HL-1 cells	lncRNA HOTAIR ↓	miR-613 ↑	Cx43 ↓	Atrial fibrillation	[63]

↑: upregulation; ↓: downregulation. iPSC-CMs, murine induced pluripotent stem cell-derived cardiomyocytes; VSMC, vascular smooth muscle cell; PASMC, pulmonary artery smooth muscle cell; EPC, endothelial progenitor cell; *CDKN2A*, cyclin-dependent kinase inhibitor 2A.

**Table 3:** Noncoding RNAs and related connexins involved in other systems.

Animal Tissue/Primary Cells	Noncoding RNAs	Molecular Target	Related Connexin	Disease/Pathology	Ref
Uteruses of mice/human myometrial smooth muscle cells	miR-212-3p ↑	MeCP2 ↓	Cx43 ↑	Myocyte contraction	[75]
Human articular chondrocytes	miR-382-3p ↓	Cx43 ↑	Cx43 ↑	Osteoarthritis	[65]
Joints of mice/human articular chondrocytes	miR-1 ↓	Cx43 ↑	Cx43 ↑	Osteoarthritis	[69]
Human chondrocytes	miR-33a-5p ↑	Sp1 ↓	Cx43 ↑	Osteoarthritis	[64]
Human osteoblasts	miR-31-5p ↑	Sp1 ↓	Cx43 ↓	Osteoarthritis	[64]
Nucleus pulposus tissue of rats/human nucleus pulposus cells	miR-206 ↓	Cx43 ↑	Cx43 ↑	Intervertebral disk degeneration	[66]
Ligament tissue of humans/human ligament fibroblast cells	lncRNA MALAT1 ↑	miR-1 ↓	Cx43 ↑	Ossification	[67]
Molars of mice/rat dental epithelial cells	miR-1 ↑	Cx43 ↓	Cx43 ↓	Cell proliferation	[70]
Human dental pulp stromal cells	circAKT3 ↑	miR-206 ↓	Cx43 ↑	Osteogenesis	[71]
Cochlea of mice	miR-34c-5p ↑	Cx26 ↓	Cx26 ↓	Hearing impairment	[74]

↑: upregulation; ↓: downregulation. MeCP_2_: methyl-CpG-binding protein 2; Sp1: specific protein (Sp)-transcription factor 1.

**Table 4:** Noncoding RNAs and related connexins involved in cancers.

Animal Tissue/Primary Cells	Cell Line	Noncoding RNAs	Molecular Target	Related Connexin	Disease/Pathology	Ref
NSCLC tissue of patients/human lung cancer cells	/	miR-613 ↓	Cx43 ↑	Cx43 ↑	Non-small cell lung cancer	[76]
Human bladder cancer cells	/	miR-139-5p ↓	Cx43 ↑	Cx43 ↑	Bladder cancer	[78]
Bladder cancer tissue of patients/human bladder cancer cells	/	miR-1298-5p ↓	Cx43 ↑	Cx43 ↑	Bladder cancer	[77]
Human hypopharyngeal squamous carcinoma cells	/	lncRNA LEF1-AS1 ↑	miR-221-5p ↓	Cx43 ↑	Hypopharyngeal squamous carcinoma	[79]
Human metastatic breast cancer cells	/	lncRNA CCRR ↑	CIP85 ↓	Cx43 ↑	Breast cancer metastasis	[81]
/	Metastasis pancreatic cancer cell M8 and its parental cell BxPC.3	lnRNA HELLPAR ↑, lncRNA OIP-AS1 ↑	hsa-miR-30d-5p ↓	Cx43 ↑	Pancreatic cancer metastasis	[80]
Ovarian cancer tissue of humans/human ovarian cancer cells	/	miR-2114-3p ↓	Cx26 ↑	Cx26 ↑	Ovarian cancer	[91]
Tumors of mice/human ovarian cancer cells	/	miR-206 ↑	Cx43 ↓	Cx43 ↓	Epithelial ovarian cancer	[82]
Ovarian cortexes of patients	/	miR-130b ↑	Cx43 ↓	Cx43 ↓	Polycystic ovary syndrome	[92]
Bone marrow aspirates and peripheral blood of patients	Leukemic and HEK293T cells	miR130a/b-3p ↑	Cx43 ↓	Cx43 ↓	Leukemia	[83]
Gastric cancer tissue of patients/human gastric cancer cells	/	miR-301-3p ↑	Cx43 ↓	Cx43 ↓	Gastric cancer	[84]
Pancreatic cancer tissue of patients/human pancreatic ductal adenocarcinoma cells	/	miR-30a-3p ↑	Cx43 ↓	Cx43 ↓	Pancreatic cancer	[86]
Pancreatic cancer tissue of patients/human pancreatic ductal adenocarcinoma cells	/	miR-30b-5p ↑	Cx43 ↓	Cx43 ↓	Pancreatic cancer	[85]
Human malignant melanoma cells, human epidermal melanocytes	/	miR-106a ↑	Cx43 ↓	Cx43 ↓	Melanoma	[88]
Human melanoma cells	/	miR-335-5p ↑	Cx31 ↓	Cx31 ↓	Melanoma	[87]

↑: upregulation; ↓: downregulation.

## Data Availability

The data used to support the findings of this study are included within the article.

## Supplementary Materials

The following supporting information can be downloaded at: https://www.mdpi.com/article/10.3390/ijms25116146/s1.
