# Supplementary material for: Perspective and Therapeutic Potential of the Noncoding RNA–Connexin Axis
Source: Int J Mol Sci. 2024 Jun 2;25(11):6146. doi: 10.3390/ijms25116146 (PMC11173347; doi:10.3390/ijms25116146)
Supplement: Supplementary file 1 [file ijms-25-06146-s001.zip › ijms-2976240-supplementary.pdf]

Table S1. Connexin genes and corresponding proteins

Human		Mouse	
Gene	Protein	Gene	Protein
GJA1	CX43	*Gja1*	Cx43
GJA3	CX46	*Gja3*	Cx46
GJA4	CX37	*Gja4*	Cx37
GJA5	CX40	*Gja5*	Cx40
GJA8	CX50	*Gja6*	Cx33
GJA9	CX59	*Gja8*	Cx50
GJA10	CX62	*Gja10*	Cx57
GJB1	CX32	*Gjb1*	Cx32
GJB2	CX26	*Gjb2*	Cx26
GJB3	CX31	*Gjb3*	Cx31
GJB4	CX30.3	*Gjb4*	Cx30.3
GJB5	CX31.1	*Gjb5*	Cx31.1
GJB6	CX30	*Gjb6*	Cx30
GJB7	CX25	/	/
GJC1	CX45	*Gjc1*	Cx45
GJC2	CX47	*Gjc2*	Cx47
GJC3	CX30.2/31.3	*Gjc3*	Cx29
GJD2	CX36	*Gjd2*	Cx36
GJD3	CX31.9	*Gjd3*	Cx30.2
GJD4	CX40.1	*Gjd4*	Cx39
GJE1	CX23	*Gje1*	Cx23

Connexin genes and their corresponding proteins are obtained from https://www.omim.org (accessed on May 20 2024) and https://www.informatics.jax.org (accessed on May 20 2024).
